# Effectiveness of Smartwatch Device on Adherence to Home-Based Cardiac Rehabilitation in Patients With Coronary Heart Disease: Randomized Controlled Trial

**DOI:** 10.2196/70848

**Published:** 2025-09-18

**Authors:** Sisi Zhang, Yuehui Wang, Jiahui Wu, Changsheng Ma, Xiaoping Meng

**Affiliations:** 1 Department of Cardiology, Beijing Anzhen Hospital, Capital Medical University, Beijing, China; 2 Department of Geriatrics, Jilin Geriatrics Clinical Research Center, The First Hospital of Jilin University, Changchun, China; 3 Department of Cardiovascular Disease, Affiliated Hospital of Changchun University of Chinese Medicine, No.1478 Gongnong Road, Chaoyang District, Changchun, Jilin Province, 130000, China, 86 13180889430

**Keywords:** smartwatch, digital technology, home-based cardiac rehabilitation, adherence, anxiety, depression, quality of life

## Abstract

**Background:**

Digital technologies have the potential to overcome many of the limitations associated with traditional center-based cardiac rehabilitation (CBCR), such as limited accessibility, transportation barriers, and low adherence. In this context, home-based cardiac rehabilitation (HBCR) has emerged as a promising alternative. However, maintaining adherence and providing continuous supervision in remote settings remain a major challenge. Smartwatch-based interventions may offer a novel solution to support and monitor patients in HBCR programs, yet robust clinical evidence is still limited.

**Objective:**

This study was designed to investigate the effectiveness of a smartwatch-facilitated HCBR model in improving exercise adherence and health-related outcomes in patients with coronary heart disease (CHD), aiming to improve adherence and other outcomes related to the secondary prevention of cardiovascular disease.

**Methods:**

We conducted a prospective, single-center, randomized, parallel-controlled, non-blinded trial. Eligible participants were adults (≥18 years) with a confirmed diagnosis of CHD, recruited from a tertiary hospital in Jilin Province, China. Participants were randomly assigned in a 1:1 ratio to either the intervention group (smartwatch-facilitated HBCR) or the control group (standard HBCR) for a duration of 3 months. The intervention group received a comprehensive program delivered via a smartwatch, including real-time feedback, remote supervision, physical activity monitoring, and educational content. The control group received conventional HBCR without technological assistance. The primary outcome was adherence to the HBCR program, assessed using the Home-Based Cardiac Rehabilitation Exercise Adherence Scale. Secondary outcomes included cardiopulmonary function (peak VO₂ measured via cardiopulmonary exercise testing), anxiety (Generalized Anxiety Disorder-7), depression (Patient Health Questionnaire-9), and health-related quality of life (36-Item Short Form Survey, SF-36), evaluated at baseline and at 3 months.

**Results:**

Between January 1 and December 30, 2023, a total of 62 patients (mean [SD] age 59.93 [10.06] years; 40.4% women [25/62]) were enrolled and randomized to the intervention group (n=32) or control group (n=30). Baseline characteristics were well balanced between the groups. At 3 months, participants in the smartwatch group demonstrated significantly higher adherence scores compared to the control group (*P*<.01). Additionally, the smartwatch group showed significant improvements in peak VO₂ (*P*<.01), anxiety (GAD-7, *P*<.01), depression (PHQ-9, *P*<.01), and selected domains of SF-36 (*P*<.05). No serious adverse events related to the intervention were reported, and user engagement with the smartwatch platform was high throughout the study period.

**Conclusions:**

This study demonstrates that a smartwatch-facilitated HBCR model is both feasible and effective in enhancing adherence and improving clinical outcomes among patients with CHD. These findings support the integration of wearable technology into routine HBCR and lay the groundwork for future large-scale, multicenter trials.

## Introduction

### Background

Coronary heart disease (CHD), as the leading cause of death globally, presents significant public health and economic challenges [1-3]. Exercise-based cardiac rehabilitation (CR), an important management strategy for CHD, is strongly recommended by clinical practice guidelines from the American College of Cardiology, American Heart Association, and European Society of Cardiology [4,5]. Despite its recognized benefits, access to these services remains limited for many patients in China due to barriers such as accessibility, time constraints, and uneven economic and technological development across regions [6]. As an alternative, home-based cardiac rehabilitation (HBCR) is similarly effective in improving clinical outcomes [7]. However, the benefits of HBCR largely depend on long-term adherence. Previous studies have reported inconsistent findings regarding patients’ adherence to HBCR [8,9].

With the development of mobile health (mHealth) technology, remote cardiac rehabilitation programs now use the capabilities of internet-connected devices, wearable monitors, smartphones, and smartwatches. These tools enable the delivery of comprehensive rehabilitation services that were previously available only through in-person sessions at medical facilities [10]. The American Heart Association has noted that mHealth technologies play a crucial role in enhancing physical activity, supporting smoking cessation, promoting weight loss, managing blood glucose levels, and controlling hypertension [11]. The World Health Organization has emphasized the role of digital health technologies in improving health care delivery, particularly for chronic disease management and rehabilitation [12]. Studies across various countries have demonstrated the effectiveness of digital platforms, mobile applications, and wearable devices in supporting CR. For instance, a study in Canada reported that tele-rehabilitation programs provided comparable outcomes to in-person CR in terms of functional recovery and patient satisfaction [13]. Additionally, mHealth technologies now allow for the near real-time monitoring of patient performance data at home. This capability covers various aspects of physical activity, such as intensity, duration, distance traveled, number of steps, and sedentary periods, as well as vital signs such as heart rate and blood pressure, thus facilitating remotely supervised HBCR programs.

Among various wearable devices, smartwatches stand out due to their ability to provide continuous, real-time monitoring of vital signs and physical activity metrics such as heart rate, steps, exercise intensity, and sedentary time. This makes them uniquely suited for remotely supervised HBCR programs, offering timely feedback and enhanced patient engagement. Despite this potential, research specifically evaluating smartwatch-facilitated HBCR remains limited, especially in China where mHealth-based HBCR programs are still in early development stages and data on patient acceptance and adherence are scarce.

In China, the development of mHealth HBCR programs is still in the nascent stages; research on patient acceptance and adherence to these mHealth-enabled HBCR programs remains sparse.

### Objectives

This randomized controlled trial was designed to assess the effectiveness of a remote HBCR program supported by a smartwatch device and to determine whether this innovative mHealth HBCR model enhances adherence to CR and improves other outcomes related to the secondary prevention of cardiovascular disease.

## Method

### Study Design and Participants

This study was a single-center, 2-arm parallel design randomized controlled trial performed in a tertiary hospital in Jilin Province, China, from January 2023 to December 2023.

### Inclusion Criteria

The inclusion criteria were as follows: (1) age of more than 18 years old; (2) diagnosed with CHD (including myocardial infarction and stable angina), with stable clinical manifestations and cardiovascular-related examination indicators; (3) a documented diagnosis of CHD and if the patient had either undergone percutaneous coronary intervention, experienced a recent myocardial infarction, or were referred for secondary prevention following a clinical evaluation. The need for cardiac rehabilitation was based on established clinical guidelines and individualized physician recommendations.

### Exclusion Criteria

The exclusion criteria included (1) unstable angina pectoris, acute myocardial infarction, uncontrolled atrial tachycardia, asthma, obstructive hypertrophic cardiomyopathy, severe pulmonary hypertension, acute myocarditis, severe valvular heart disease, and other conditions where cardiopulmonary exercise testing is contraindicated (nerve, skeletal muscle, and rheumatic diseases); (2) uncontrolled hypertension (systolic blood pressure >160 mmHg or diastolic blood pressure  >100 mmHg at rest); (3) uncontrolled heart failure (New York Heart Association class III-IV); (4) have severe motor, cognitive, mental illnesses, or visual impairment limiting the smartphone and smartwatch use; (5) high‐risk for falls; and (6) being unwilling or unable to provide informed consent.

### Randomization

The participants enrolled were randomized 1:1 to receive the smartwatch device-based CR or usual care CR alone. Participants were assigned to either the control or the intervention group at a ratio of 1:1 through computerized randomization. Due to the nature of the intervention, neither the participants nor the outcome assessors were blinded to the group assignments.

Care providers involved in the study were assigned based on their roles to ensure consistency and minimize potential bias. A dedicated team of cardiac rehabilitation specialists and research staff was responsible for participant enrollment, baseline assessments, and follow-up evaluations. To maintain protocol adherence, health care providers delivering the intervention were specifically trained in the use of the smartwatch-based rehabilitation program, while those managing the control group followed standard cardiac rehabilitation guidelines. Importantly, the care providers administering the intervention did not overlap with those assessing outcomes to reduce potential bias. Additionally, all care providers were blinded to the study hypotheses and instructed to follow standardized procedures for patient interactions.

### Control Group

Participants in the control group received a standard HBCR program based on the secondary prevention guidelines [14].

### Intervention Group

**Figure 1.**
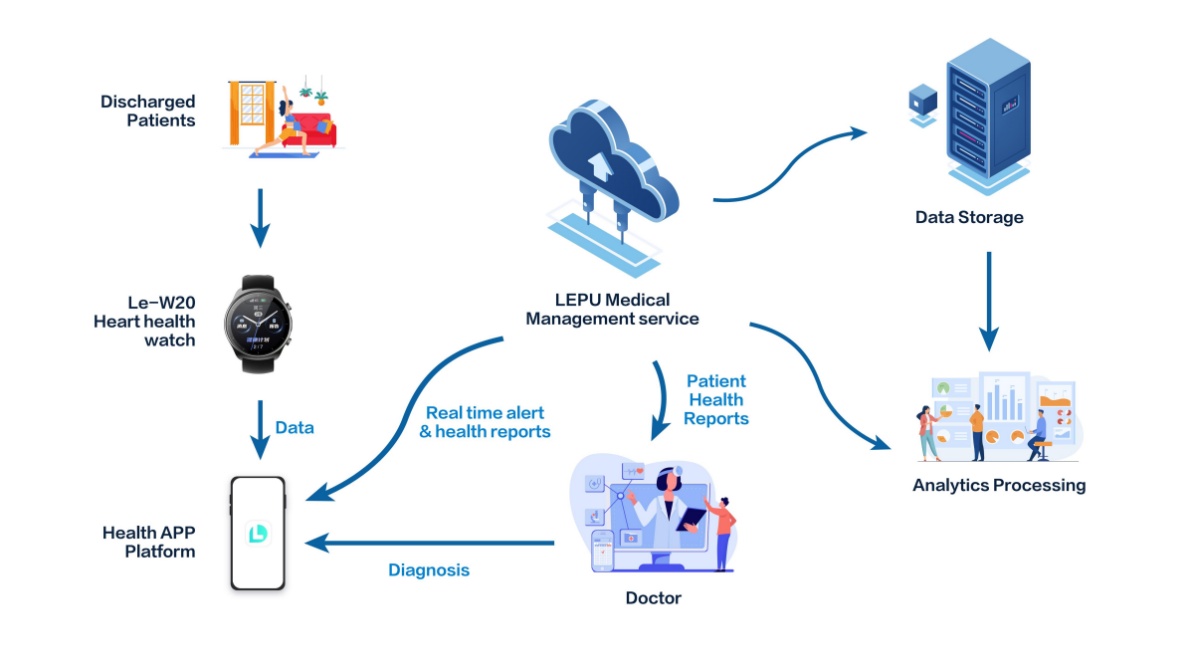
Diagram showing the flow of data for the intervention group.

The smartwatch-based HBCR program was delivered through a smartphone app. At the time of discharge, participants in the intervention group were provided with a smartwatch pre-installed with the intervention application. Additionally, they were instructed to download the corresponding mobile app onto their personal smartphones via the designated app stores. A step-by-step user guide was provided, and trained health care staff conducted a one-on-one demonstration to ensure participants could successfully log in, navigate the interface, and use key features such as exercise tracking, medication reminders, and data sharing. Technical support was available through a dedicated helpline to assist with any issues related to app access or usage during the study period. The intervention consists of real-time monitoring, automatic reminders, health education, data sharing, and individualized follow-up (Figure 1).

#### Real-time Monitoring

With high-precision sensors, the smartwatch continuously tracks the patient’s heart rate, blood pressure, and blood oxygen levels during exercise. Furthermore, the smartwatch could calculate the number of calories burned during the exercise session, helping patients understand their energy expenditure and adjust their exercise intensity accordingly. This comprehensive real-time monitoring enables both patients and health care providers to track progress and make informed decisions to optimize the effectiveness and safety of the HBCR program.

#### Automatic Reminders

Specific prescriptions set by the system are transmitted to the patient’s smartwatch, providing daily reminders at designated times. For example, the doctor inputs the medication prescription into the system and sets reminder times. At 8 AM each day, the patient receives a reminder to take antihypertensive medication, including the specific name, dosage, and other important instructions. At 8 PM each evening, the smartwatch reminds the patient to take their statin medication (such as simvastatin, atorvastatin, rosuvastatin, or pravastatin) with the specific dosage. Additionally, every Monday, Wednesday, and Friday (or at patient-chosen intervals), the patient receives a notification of their exercise plan for the day, based on their exercise prescription. At 6 AM each day, the patient is reminded of their daily hydration goal, such as drinking 1500‐2000 ml of water. If the smartwatch shuts down due to low battery or other reasons, it will send any missed reminders once it is powered back on. After the patient completes a task, they can mark it as “Completed,” which will automatically be sent into the hospital information system.

#### Health Education

After enrollment, the eligible participants will receive app-based messages regarding dietary habits, physical activity, prevention of hypertension, diseases, cardiovascular disease, and knowledge of smoking cessation. The app also included educational resources for symptom management and other CR-related topics in the form of text, images, and videos in the native language.

#### Data Sharing

Each day, we share with patients and their caregivers a summary of their motion data via the app installed on their smartphones. By sharing these results, the health care providers give feedback, which is then sent to the caregivers for supervision and guidance.

#### Individualized Follow-Up

The app would pop up the goal achievement according to pre-set intervals automatically. The participants had to reply depending on whether they had achieved the tasks or not, in addition to the Borg scale score to evaluate the exercise intensity.

### Data Collection, Outcomes, and Measures

Data were collected at baseline and 3 months. The demographic and clinical data were collected from questionnaires or electronic medical records.

The primary outcome measure was HBCR adherence as assessed using the home-based exercise training adherence questionnaire (HETAQ), which is composed of 17 items across the following domains: program comprehension, patient motivation, family support, and peer support. The total score ranged from 0 to 100 (higher scores indicating greater HBCR adherence), and ≥60 was considered to adhere well to the HBCR program [15].

Secondary outcomes included exercise capacity, quality of life, and psychological distress. Exercise capacity was measured using the cardiopulmonary exercise testing (CPET). The parameters obtained from the CPET, including oxygen consumption (VO_2_), minute ventilation (VE), carbon dioxide output (VCO_2_), peak metabolic equivalents (METs), peak VE/VCO_2_ ratio, ventilatory efficiency (VE/VCO_2_ slope), work efficiency (deltaVO_2_/deltaWR), O_2_ plus, and anaerobic threshold (AT).

The quality of life was assessed with the 36-item Short-Form Health Survey(SF-36), with the score ranging from 0 to 100, and higher scores reflect better quality of life [16]. Depression and anxiety symptoms were measured using the Patient Health Questionnaire 9-item scale (PHQ-9) and the Generalized Anxiety Disorder 7-item scale (GAD-7) [17,18]. Scores on the PHQ-9 range from 0 to 27, with results of no depression, or mild, moderate, moderately severe, and severe depression. Scores on the GAD-7 range from 0 to 21 points, with result of mild, moderate, and severe levels of anxiety symptoms. Higher points on these scales were closely related to psychological distress.

### Sample Size

Sample size calculation was performed using the results of the preliminary study, which showed that the mean (SD) HETAQ score was 85.99 (6.68) in the intervention group versus 72.68 (12.93) in the control group. With the 95% confidence interval, 80% power, 5% type I error, and 10% dropout rate, we determined a minimum of 16 participants per group at recruitment. The calculation was based on two independent *t* tests, using PASS, version 15 (NCSS LLC).

### Statistical Analysis

Data were analyzed using the intention-to-treat principles using the Statistical Package for Social Sciences software (SPSS, version 26.0). Statistical analyses were conducted by an independent statistician who was blinded to group assignments. The dataset was anonymized, with group labels concealed during statistical processing to prevent bias in interpretation.

The statistical analysis followed the intention-to-treat principle, meaning all randomized participants were included in their originally assigned groups, regardless of adherence to the intervention. Participants who remained in the trial but did not use the application as prescribed were still included in the analysis. Their adherence data were categorized separately in exploratory analyses to assess potential differences in outcomes based on actual app usage.

Baseline comparisons were performed using descriptive statistics. The categorical variables were summarized as frequencies and percentages, and the quantitative variables were expressed as means (standard deviation) or medians (interquartile range) depending on the distribution of the values. Before conducting group comparisons, all continuous variables were assessed for normality using the Shapiro-Wilk test. Variables conforming to a normal distribution were analyzed using independent-sample *t* tests for between-group comparisons and paired-sample *t* tests for within-group comparisons. For variables that did not meet the normality assumption, non-parametric tests such as the Mann-Whitney *U* test (between groups) and Wilcoxon signed-rank test (within groups) were applied. Within-group analyses compared pre- and post-intervention outcomes to evaluate changes over time in each group separately. Between-group analyses at follow-up assessed the differences in outcomes between the intervention and control groups.The *P* value ≤.05 was considered significant.

### Ethical Considerations

This study protocol adhered to the Declaration of Helsinki and received approval from the institutional review board (IRB) of the Affiliated Hospital of Changchun Traditional Chinese Medicine (approval no. CCZYFYKYLL2023-164). All participants provided written informed consent before enrollment in the study. The consent process ensured that participants were fully informed about the study’s purpose, procedures, potential risks, and benefits.

To safeguard user privacy and ensure data security, each participant is required to log into the device using a unique account and password. Collected data are anonymized before being uploaded to a secure cloud server in full compliance with advanced data protection standards.

All participants who completed the trial received appropriate compensation for their time and commitment. Specifically, each participant was allowed to retain the smartwatch device used during the study, which has an estimated retail value of approximately US $200. No additional monetary compensation was provided. This non-cash compensation was disclosed during the informed consent process and was provided equally to participants in both the intervention and control groups to ensure fairness and transparency.

## Results

### Overview

**Figure 2.**
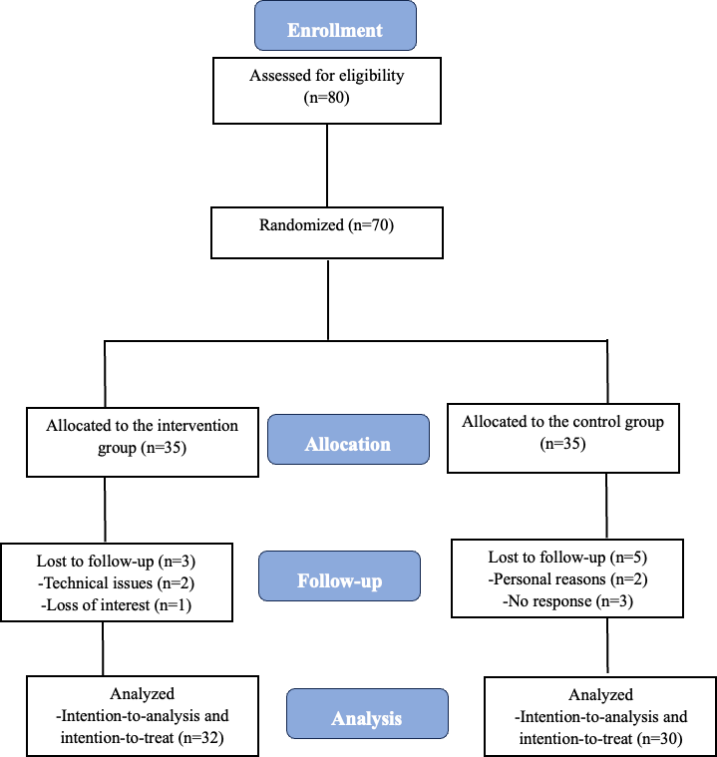
CONSORT diagram of the flow of participants.

Figure 2 illustrates the CONSORT diagram of the flow of participants (CONSORT-eHEALTH checklist is provided in Checklist 1). A total of 70 eligible participants were randomly assigned to the smartwatch group (n=35; intervention group) or usual care (n=35). During the follow-up period, 3/35 participants dropped out in the intervention group and 5/35 dropped out in the control group. In the intervention group, 2 participants withdrew due to technical difficulties with the application, and 1 due to loss of interest. In the control group, 3 participants were lost to follow-up, and 2 withdrew due to personal reasons unrelated to the study.

Table 1 shows the baseline characteristics of patients included in this study. There were no significant differences between the two groups at baseline considering the sociodemographic and clinical variables. The overall mean (SD) age of the participants in the intervention and control groups was 59.93 (10) years and 62.67 (4.89) years, respectively.

**Table 1.** Baseline characteristics.

Variable	Total (n=62)	Intervention group (n=32）	Control group (n=30)	Chi-square test (*df*)	Two-tailed *t* test *(df)*	*P* value
Gender, n (%)				0	—^a^	>.99
Male	37 (59.6)	18 (56.2）	19 (63.3）			
Female	25 (40.4)	14 (43.8）	11 (36.7）			
Age (y), mean (SD)	59.93 (10.06)	62.67 (4.89)	57.2 (13)	—	1.52 (37)	.14
Height (cm), mean (SD)	167.3 (7.25)	167.07 (6.96)	167.53 (7.76)	—	−0.17 (58)	.86
Weight (kg), mean (SD)	69.4 (14.1)	69.3 (17.89)	69.5 (9.58)	—	−0.03 (48)	.97
BMI (kg/m^2^), mean (SD)	25.47 (3.96)	25.97 (3.9)	24.9 (4.08)	—	0.67 (59)	.5
Marital status, n (%)				1.034 (1)	—	>.99
Married	60 (96.8)	32 (100）	28 (93.3）			
Single, divorced, or windowed	2 (3.22)	0 (0）	2 (6.7）			
Employment status, n (%)				1.64 (1)	—	.44
Retired	20 (32.3)	10 (31.2）	10 (33.3）			
Employed	32 (51.6)	18 (56.2）	14 (46.7）			
Others	10 (16.1)	4 (12.5）	6 (20）			
Medicine, n (%)						
Antiplatelets	58 (93.5)	28 (87.5）	30 (100）	1.15 (1)	—	.59
Statins	42 (67.7)	18 (56.2）	24 (80）	1.29 (1)	—	.45
β-blocker	4 (6.5)	2 (6.25）	2 (6.7）	0 (1)	—	1
ACEI^b^ or ARB^c^	16 (25.8)	10 (31.3）	6 (20）	1.67 (1)	—	.39
CCB^d^	12 (19.4)	6 (18.8）	6 (20）	0.18 (1)	—	1
White blood cell count (×10^9^/L), mean (SD)	5.76 (1.11)	5.56 (1.04)	5.94 (1.18)	—	−0.91 (58)	.36
Red blood cell count (×10^12^/L), mean (SD)	4.68 (0.69)	4.43 (0.49)	4.91 (0.77)	—	−1.99 (46)	.06
Platelet count (×10^9/^L), mean (SD)	210.48 (65.4)	196.5 (85)	223.53 (38.2)	—	−1.11 (38)	.27
Hemoglobin level (g/L), mean (SD)	149.72 (38.35)	157.64 (52.73)	142.33 (15.44)	—	1.07 (31)	.29
Low-density lipoprotein level (mmol/L), mean (SD)	2.66 (0.78)	2.6 (0.76)	2.71 (0.82)	—	−0.38 (58)	.7
Total cholesterol level (mmol/L), mean (SD)	4.51 (0.8)	4.32 (0.82)	4.69 (0.77)	—	−1.27 (57)	.21
High-density lipoprotein level (mmol/L), mean (SD)	1.16 (0.29)	1.1 (0.21)	1.21 (0.34)	—	−1.0 (50)	.30
Triglyceride level (mmol/L), mean (SD)	1.54 (0.72)	1.59 (0.85)	1.49 (0.58)	—	0.39 (46)	.69
Blood urea nitrogen level (mmol/L), mean (SD)	6.17 (2)	6.49 (2.5)	5.86 (1.29)	—	0.86 (43)	.39
Serum creatinine level (μmol/L), mean (SD)	82.4 (29.99)	84.6 (40.85)	80.2 (13.59)	—	0.39 (35)	.69
Blood glucose level (mmol/L), mean (SD)	6.15 (1.75)	6.48 (2.39)	5.81 (0.63)	—	1.04 (31)	.3
Left ventricular measurement (mm), mean (SD)	47.12 (4.67)	47.29 (5.95)	46.92 (2.74)	—	0.19 (60)	.84

a not applicable.

b ACEI: angiotensin-converting enzyme inhibitor.

c ARB: angiotensin II receptor blocker.

d CCB: calcium channel blocker.

### Primary Outcome

At 3 months, the mean (SD) HETAQ score was 89.17 (9.29) for the intervention group and 72.50 (17.17) for the control group (*P*=.003). However, there was no overall significant difference between the groups in the domains of peer support (*P*=.311; Table 2).

**Table 2.** HETAQ^a^ score (mean, SD) differences between the groups at 3 months.

Category	Patient’s perception	Patient’s motivation	Family support	Peer support	Total score
Intervention group (n=32)	90.66 (12.91)	88.33 (11.70)	75.56 (9.65)	56.94 (13.42)	89.17 (9.29)
Control group (n=30)	50.83 (19.17)	58.05 (22.41)	59.00 (18.75)	50.83 (18.58)	72.50 (17.17)
Two-tailed *t* test *(df)*	7.68 (50)	4.639 (43)	3.041 (43)	1.033 (53)	3.307 (44)
*P* value	<.001	<.001	<.001	.311	<.001

a HETAQ: home-based exercise training adherence questionnaire.

### Secondary Outcomes

Regarding the CPET parameters, only the peak VO_2_ differed within and between groups postintervention. After intervention, the mean (SD) peak VO_2_ of the intervention group (19.8 [4.3]) was significantly superior to that of the control group (15.88 [2.3], *P*<.05; Table 3).

**Table 3.** Comparing cardiopulmonary exercise testing (CPET) parameters at baseline and at 3 months after the intervention.

CPET	Intervention group (n=32）		*P* value^a^	Control group (n=30)		*P* value^a^	*P* value^b^	*P* value^c^
	Baseline	3 months		Baseline	3 months			
Peak VO_2_^d^ (ml/kg/min), mean (SD)	15.66 (3.3)	19.8 (4.3)	.002	16 (1.6)	15.88 (2.3)	.91	.81	<.001
Peak Mets^e^, mean (SD)	4.37 (0.64)	5.17 (0.55)	.000	4.88 (0.74)	5.55 (1.7)	.23	.21	.55
Peak O_2_ plus (ml/beat), mean (SD)	9.46 (2.47)	10.41 (2.59)	.004	10.15 (1.94)	9.2 (2.11)	.47	.58	.36
Peak ventilation (ml/kg/min), mean (SD)	35.51 (5.74)	38.58 (6.88)	.192	39.71 (7.96)	41.32 (9.52)	.56	.27	.54
AT^f^ (ml/kg/min), median (IQR)	11.5 (10.1-12.85)	13.6 (11.95-14.9)	.012	12.4 (5.88-13.65)	12.4 (11.35-16.4)	.75	.73	.84
VE/VCO_2_^g^, median (IQR)	26.61 (23.48-30.33)	26.18 (24.2-28.25)	.069	25.32 (22.39-25.98)	23.43 (20.59-25.94)	.11	.12	.12
△VO_2_/△WR^h^ (ml/min/w), mean (SD)	8.31 (1.58)	8.71 (1.54)	.422	8.28 (1.38)	9.11 (2.53)	.29	.97	.71

a *P* value derived from test T1 (3 months) vs T0 (baseline).

b *P* value derived from the test T0 intervention group vs T0 control group.

c *P* value derived from the test T1 intervention group vs T1 control group.

d VO_2_: oxygen consumption.

e Mets: metabolic equivalent of tasks.

f AT: anaerobic threshold.

g VE/VCO_2_: ventilatory equivalent for carbon dioxide.

h △VO_2_/△WR: functional gain or the increase in VO_2_ per W.

**Figure 3.**
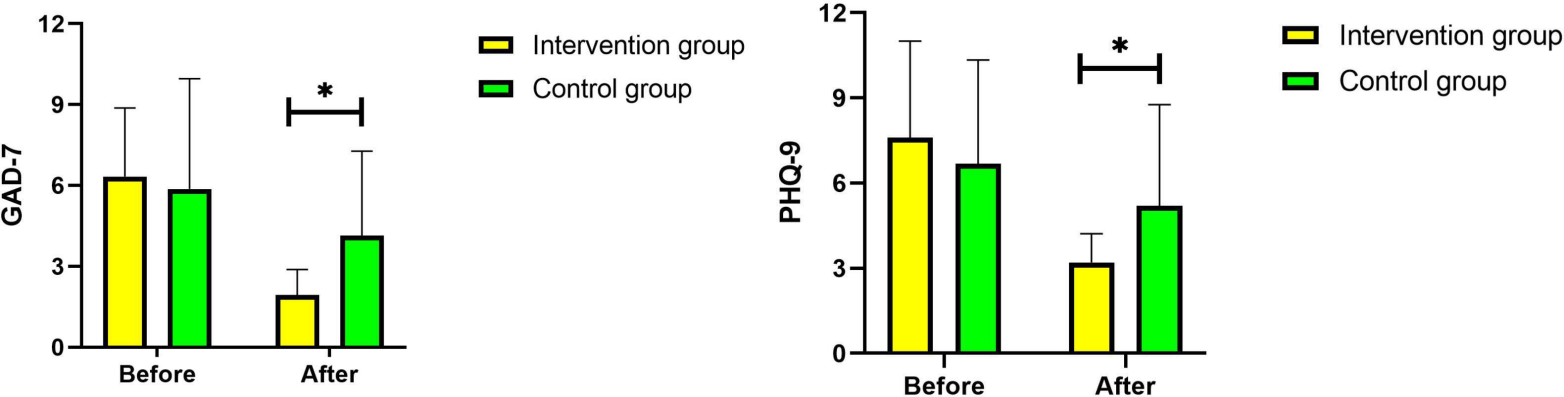
Changes in the Generalized Anxiety Disorder 7-item (GAD-7) scale and Patient Health Questionnaire 9-item (PHQ-9) scale scores before and after intervention.

Changes in PHQ-9 and GAD-7 scores in the two groups are shown in Table 4. At baseline, there was no significant difference in the mean PHQ-9 and GAD-7 scores between the two groups (*P*>.05). After the 3-month intervention, the mean PHQ-9 and GAD-7 scores of the intervention group were lower than those at baseline (*P*<.05), whereas the scores for the control group showed no differences over the same period Figure 3.

**Table 4.** Comparison of anxiety, depression, and quality of life between the two groups before and after intervention.

	Intervention group (n=32）		Control group (n=30）	
	Baseline	3 months	Baseline	3 months
Anxiety and depression				
PHQ-9^a^ score, mean (SD)	7.6 (3.4)	3.2 (1.01)^bc^	6.67 (3.66)	5.2 (3.57)
GAD-7^d^ score, mean (SD)	6.33 (2.55)	1.93 (0.96)^bc^	5.87 (4.09)	4.13 (3.15)
Quality of life (SF-36)				
Physical function, median (IQR)	90 (90-95)	95 (90-95)	90 (90-93.75)	95 (90-95)
Bodily pain, median (IQR)	52 (0-87.5)	62 (49-74)^bc^	0 (0-68.75)	80 (80-88.5)
Social function, mean (SD)	67.52 (13.95)	61.53 (17.34)^bc^	69.44 (10.72)	83.61 (11.69)
General health, median (IQR)	45 (40-52)	50 (43-52)^b^	45 (41.25-50)	52 (45-60.75)
Energy, mean (SD)	52.31 (17.27)	62.46 (12.02)^bc^	52.08 (12.33)	72.25 (9.23)
Physical role, median (IQR)	50 (0-87.5)	25 (25-62.5)^bc^	0 (0-68.75)	100 (75-100)
Emotional function, median (IQR)	33.33 (16.66-66.67)	33.33 (16.66-66.67)^b^	33.33 (0-58.33)	83.33 (33.33-100)
Mental health(MH), mean (SD)	53.23 (20.35)	69.23 (17.46)^b^	59.67 (12.92)	78.33 (7.71)
Health transition, median (IQR)	25 (25-50)	36 (25-75)^bc^	25 (25-43.75)	75 (50-75)
Total score, mean (SD)	971.29 (116)	523.51 (69.72)^bc^	1087.64 (93.57)	678.3 (62.48)^b^

a PHQ-9: Patient Health Questionnaire 9-item scale

b Compared with the same group after intervention, *P*<.05.

c Compared with the control group after intervention, *P*<.05.

d GAD-7: Generalized Anxiety Disorder 7-item scale.

The mean (SD) SF-36 scores of the intervention and control groups at baseline were 971.29 (116) points and 1087.64 (93.57) points, respectively. The scores did not differ significantly between the two groups (*P*>.05). After the intervention, the scores for the following domains (bodily pain, social function, general health, energy, physical role, emotional function, mental health, and health transition) improved significantly in the intervention group, whereas the control group showed no change. The between-group changes after intervention showed significant differences between the bodily pain, social function, energy, physical role, and health transition domains (Table 4).

## Discussion

### Principal Findings

Our findings suggest that the smartwatch-assisted HBCR program may help improve cardiac rehabilitation adherence and peak VO₂, with potential benefits in quality of life, depression, and anxiety symptoms. These improvements, although statistically significant, should be interpreted with caution given the modest sample size and short follow-up duration. Nonetheless, the high acceptance and engagement rates indicate that the proposed wearable device is well tolerated and feasible for use in HBCR programs, supporting its potential for broader clinical application.

Recently, the development of wearable devices has attracted attention given the advances in health care. Previous studies on the technology of HBCR have mainly included telephone, smartphone applications, and remote monitoring systems [19-21]. We have provided evidence that patients who underwent comprehensive HBCR based on the smartwatch application demonstrated better adherence and improved peak VO_2_ after 3 months.

Non-adherence to HBCR is a major problem, especially for women, older adults, and ethnic minority populations [22]. As previous studies reported, unlike the center-based CR, the adherence for HBCR was usually in the lower range [23]. The adherence to HBCR is mainly determined by external factors, such as environmental factors, social factors, transportation factors, and patient-self factors. We believe that the smartwatch’s feature of automatic reminder function has led to changes in patients’ exercise behavior, thereby improving their adherence to physical activity. The reminder function has been reported to encourage patients’ behavior toward better adherence [24].

One of the unique features of the intervention described here was the use of a commercially available smartwatch device to monitor participants’ real-time ECG, heart rate, oxygen saturation, physical activity, energy consumption, exercise time, and sleep-related parameters. Indeed, such observations were helpful for patients to manage their activity levels and allow health care providers to tailor the individual intensity of exercise through their performance. One of the major advantages of digital health interventions is their ability to deliver care remotely, which is particularly beneficial for patients who may face geographical, mobility, or time-related barriers to attending traditional, in-person rehabilitation programs. The convenience of participating in rehabilitation from home has been shown to increase program participation rates and improve patient satisfaction [13]. This accessibility can be especially important in regions where health care resources are limited or for patients with chronic conditions who find frequent hospital visits challenging.

In particular, individualized follow-up led the patients to continue the exercise training and medicine therapy and thus resulted in better adherence in the intervention group. Convenience is another reason for achieving better adherence among the intervention group; it is more convenient to access the smartwatch rather than the smartphone or other wearable devices. However, in this study, we observed no overall significant difference between the intervention and control groups in the peer support domain of the HETAQ scale. This finding suggests that while digital health interventions can enhance self-management and adherence to treatment, their impact on social support may be limited in the absence of structured peer interaction components. Unlike structured cardiac rehabilitation programs that facilitate in-person interactions, our intervention was primarily technology-driven, with limited direct peer engagement. While participants had access to digital educational resources and self-monitoring tools, the intervention did not include dedicated features for fostering peer interaction, which may explain the lack of significant improvement in this domain. Future iterations of the intervention may benefit from incorporating virtual community support or group-based engagement features.

Peak oxygen uptake (peak VO_2_), considered the gold standard for assessing aerobic fitness, is an important prognostic factor in patients with CHD. In our study, we observed a significantly greater improvement in peak VO_2_ in the intervention group at 3 months than in the control group. Our findings are similar to a previous study, which reported that wearable monitoring devices significantly improved the peak VO_2_ compared with interventions not using wearable devices [25]. As suggested by a large observational study, a 3.5 ml/kg/min increase in exercise capacity has been associated with an improvement in survival of ≈12% [26]. The increase in the smartwatch group is probably of clinical relevance, and if this improvement could be sustained over time, digital remote HBCR will likely lead to improved patient outcomes.

In our study, the smartwatch intervention also improved anxiety, depressive symptoms, and the quality of life. Depression and anxiety are very common in patients with coronary artery disease and are predictors of poorer outcomes. Additionally, improving quality of life is also a key component in the secondary prevention of coronary artery disease [27]. Notably, the European Society of Cardiology has recommended the use of e-health solutions, including mobile apps, to enhance psychosocial health as part of secondary prevention for patients with coronary artery disease [28]. However, some studies demonstrate that remote digital CR does not seem to improve patients’ psychological status. A recent meta-analysis, which included 20 studies and a total of 4535 patients, found improvements in both the physical and mental dimensions of quality of life in the eHealth group. However, the differences in overall scores did not achieve statistical significance (*P*=.15) [28]. In this study, the personalized, interactive nature of the smartwatch program may have contributed to improved mental health by promoting engagement in regular physical activity and enhancing the patient’s sense of social connectedness and support. Additionally, educational resources and real-time guidance may have provided patients with coping mechanisms to deal with the emotional challenges associated with their condition.

### Limitations

Despite the promising findings of this feasibility study, several limitations should be considered when interpreting the results. First, the single-center recruitment from Jilin Province (China) may introduce geographic selection bias, as participants shared similar cultural and socioeconomic profiles (ie, urban residents with access to smart devices). Rural populations were underrepresented, potentially inflating adherence rates compared to regions with digital literacy gaps.This selection bias could affect the applicability of the smartwatch-facilitated HBCR model in more diverse settings. Future multi-center studies involving diverse populations would help validate the applicability of the smartwatch-facilitated HBCR model across different settings. Second, the study did not account for potential confounding factors that could have influenced the outcomes, such as patients’ prior experience with digital health technologies, socio-economic status, or comorbidities. These factors may affect both adherence to the HBCR program and the patient’s overall health outcomes. A more comprehensive analysis considering such confounders could offer deeper insights into the factors influencing the success of smartwatch-based rehabilitation. Third, in China, family involvement in health care decisions is often more pronounced than in many Western countries, which could have influenced patient adherence to the intervention. Furthermore, the acceptance and familiarity with digital health technologies vary across cultures. While digital health solutions are increasingly popular in urban areas of China, older adults or those in rural regions may face challenges in using such technologies. These factors may limit the generalizability of our findings to other populations. Lastly, the duration of the intervention (3 months) may be too short to fully evaluate the long-term benefits of the smartwatch-based HBCR program. Further research with extended follow-up periods is needed to assess the sustainability of the observed improvements in cardiopulmonary function, anxiety, depression, and quality of life beyond the immediate post-intervention period.

### Conclusions

This study demonstrates that a smartwatch-facilitated HBCR model is both feasible and effective for patients with CHD. The intervention significantly improved exercise adherence, cardiopulmonary function, and psychosocial health outcomes, highlighting its potential as an accessible and scalable alternative to traditional center-based cardiac rehabilitation programs. These findings underscore the value of mHealth technologies in overcoming barriers to rehabilitation participation, promoting patient engagement, and improving overall health outcomes. Future studies with larger sample sizes and longer follow-up periods are warranted to further validate these results and explore the broader implications of digital health interventions in cardiac care.

## Supplementary material

10.2196/70848 Checklist 1 CONSORT e-HEALTH checklist (V 1.6.1).

## Abbreviations

AT: anaerobic threshold
CEPT: cardiopulmonary exercise testing
CHD: coronary heart disease
CR: cardiac rehabilitation
GAD-7: Generalized Anxiety Disorder 7-item scale
HBCR: home-based cardiac rehabilitation
HETAQ: home-based exercise training adherence questionnaire
MET: peak metabolic equivalent
PHQ-9: Patient Health Questionnaire 9-item scale
VCO_2_: carbon dioxide output
VE: minute ventilation
VE: minute ventilation
VO_2_: oxygen consumption
